# Cerebrospinal fluid L-lactate as a diagnostic marker for infectious-inflammatory disorders in the central nervous system of cattle

**DOI:** 10.3389/fvets.2024.1466920

**Published:** 2024-10-09

**Authors:** Sara Ferrini, Giulia Cagnotti, Ugo Ala, Eleonora Avilii, Claudio Bellino, Elena Biasibetti, Giuliano Borriello, Cristiano Corona, Giorgia Di Muro, Giulia Iamone, Barbara Iulini, Marzia Pezzolato, Elena Bozzetta, Antonio D’Angelo

**Affiliations:** ^1^Department of Veterinary Sciences, University of Turin, Turin, Italy; ^2^Istituto Zooprofilattico del Piemonte Liguria e Valle d'Aosta, Turin, Italy

**Keywords:** central nervous system infections, bovine neurology, cerebrospinal fluid, L-lactate, biomarker

## Abstract

**Introduction:**

Bacterial infection of the central nervous system (CNS) poses a clinical challenge and is a leading cause of neurological disorders in cattle. Human studies have demonstrated an increase in cerebrospinal fluid (CSF) L-lactate levels in bacterial meningitis. The aims of this study were to establish a Reference Interval (RI) for L-lactate in bovine CSF and assess its potential as a biomarker for detecting infectious-inflammatory disorders.

**Methods:**

CSF L-lactate was measured in the field using a commercially available lactate monitor. The RI for CSF L-lactate was calculated on healthy animals; univariate and receiver operating characteristic (ROC) analyses were performed to disclose an association between CSF L-lactate levels and interpretation of CSF in sick animals.

**Results:**

Twenty-seven healthy cattle and 86 sick cattle with either CNS infectious-inflammatory disorders (47/86) or CNS disorders of other etiology (39/86) were included in this prospective study. The RI for CSF L-lactate was 1.1–2.4 mmol/L. The concentration was higher in the cattle with neutrophilic pleocytosis and the area under the ROC curve was 0.92 compared to other animals. Based on a cut-off of 3.15 mmol/L, CSF L-lactate had diagnostic sensitivity and specificity for neutrophilic pleocytosis of 93 and 80%, respectively.

**Discussion:**

This is the first study to determine a RI for CSF L-lactate in cattle. Elevated CSF L-lactate levels indicated neutrophilic pleocytosis, which is often manifested in acute bacterial infection. The present findings may aid in diagnosis and correct use of antimicrobial drugs.

## 1 Introduction

Bacterial infection of the central nervous system (CNS) poses a clinical challenge and is a leading cause of neurological disorders in cattle (1, 2). Prompt antibiotic therapy can improve outcomes in cattle with bacterial meningoencephalitis/myelitis and reduce morbidity and mortality (3). Cerebrospinal fluid (CSF) analysis in the diagnosis of infection usually shows a moderate to marked increase in total nucleated cell count (TNCC) and total microprotein (TP) concentration which are not specific for CNS infectious diseases, however (2, 3). Furthermore, etiological diagnosis of bacterial infection of the CNS currently relies on the identification of bacteria by Gram staining or isolation in CSF culture (4), which is burdened by delayed diagnosis and inadequate sensitivity (5–7). The significance of CSF L-lactate levels as a valuable supplementary diagnostic test for acute CNS inflammation induced by bacterial infection in humans has gained recognition. Numerous studies have demonstrated an increase in CSF L-lactate levels during bacterial meningitis in the attempt to establish a cut-off (range, from 2.1 to 4.44 mmol/L) that can differentiate bacterial meningitis from other neurological disorders (8–11). Animal studies have investigated the diagnostic value of CSF L-lactate in neurological diseases in dogs (12–17) but the exact relationship between CSF L-lactate and bacterial infection in this species remains to be elucidated. A handheld lactate monitor has recently been validated for quantifying L-lactate in canine blood and CSF and a CSF reference interval (RI) has been established for this analyte (1.0–2.5 mmol/L) (18) in dogs. To date, only one published study has investigated the utility of CSF L-lactate as a diagnostic biomarker in cattle, but it failed to distinguish between bacterial infections and infectious-inflammatory disorders of different etiology (19). With the present study we wanted to explore the potential of CSF L-lactate as a field diagnostic tool for identifying CNS disorders in cattle. To do this, we thought it important to establish a RI for L-lactate in healthy bovine CSF and to determine whether CSF L-lactate can serve as a valid biomarker for detecting infection-inflammation in cattle with neurological disorders. Our hypothesis was that elevated CSF L-lactate levels may indicate acute CNS inflammation induced by bacterial infection and differentiate it from other neurological disorders.

## 2 Materials and methods

The study population was divided into two groups: a healthy group and a sick group. CSF analysis, including measurement of L-lactate, was an inclusion criterion. Blood L-lactate levels were analyzed when available but were not required for inclusion in the study.

### 2.1 Healthy group

This group included healthy cattle housed at the Department of Veterinary Science of Turin (Authorization No. 242/2020 – PR). The subjects’ health was based on an unremarkable general physical examination and normal complete blood panel. Blood and CSF samples were collected in the field.

### 2.2 Sick group

A prospective study was conducted between April 2019 and June 2023. All cattle referred for neurological signs suggestive of a CNS disorder to the Neurology Service of the Veterinary Teaching Hospital (VTH), Department of Veterinary Science of Turin were considered for inclusion in the study. All underwent general physical examination, neurologic examination by a board-certified neurologist (ADA), blood and CSF sampling in the field, and necropsy when performed. Only cases with a confirmed clinical diagnosis of CNS disorder were included; the final diagnosis was based on signalment, neurological examination, blood and CSF analysis, response to treatment, and histopathology when performed. Cases were categorized by the VITAMIN D mnemonic (20). For infectious-inflammatory conditions, we differentiated between cases with a confirmed etiological diagnosis (by post-mortem bacterial isolation from tissues or positive CSF bacterial culture) and cases with a suspected etiological diagnosis, based on clinical suspicion, informed by factors such as clinical presentation (e.g., neurolocalization), laboratory findings (type of CSF pleocytosis), and response to specific treatment.

This prospective study was conducted in accordance with current animal welfare regulations (Italian Legislative Decree 146/2001 of 26 March 2001 implementing the European Council Directive 98/58/EC of 20 July 1998). The study was approved by the Bioethics Committee of the University of Turin (Prot. N. 0622751). Written informed consent was obtained from the animal owners before veterinary assessment and treatment of their animals. Samples were collected during routine diagnostic evaluation.

### 2.3 Collection and analysis of blood and CSF samples

Blood samples (5 mL aliquots) were obtained from the jugular vein and placed into 2 tubes containing EDTA and a coagulation activator. CSF was collected from the lumbosacral region with the animal in either sternal recumbency or standing position, as described by Mayhew (21). When necessary, an animal was sedated prior to the procedure by intravenous administration of xylazine hydrochloride (Rompun, Bayer S.p.A) at a dose of 0.05 mg/kg body weight. The collection site (5 cm x 10 cm) was shaved and surgically prepared; local anesthesia was administered by subcutaneous injection of 2.5 mL of procaine hydrochloride (Procamidor®, Richter Pharma AG). CSF was collected using spinal needles (18 G and 90 mm in length or 21 G and 50 mm) (Terumo) depending on the animal’s body size. A minimum of 5 mL of CSF was collected by attaching the syringe to the hub of the needle and applying gentle aspiration. The sample was then placed in empty sterile tubes. The L-lactate concentration was measured immediately after collection of blood and CSF samples using a portable StatStrip Xpress© analyzer (Nova Biomedical). A drop of blood or CSF (approximately 0.6 μL) was applied to the free end of the test strip inserted into the analyzer and the result was read out by the device within 13 s. The samples were kept refrigerated (4°C) until transferred to the Laboratory Service of the VTH of Turin for analysis within one hour of collection, as previously described (22).

### 2.4 Statistical analysis

Statistical analysis was performed using Rstudio version 4.1.3 and Prism version 9.1.1. Numerical variables were analyzed for normality distribution with the Shapiro Wilk test and are expressed as mean and standard deviation ( ± SD) or median and interquartile range (IQR), as appropriate. Categorical variables are expressed as relative frequency and percentage. The RI with 90% Confidence Interval (CI) for CSF and blood L-lactate in the healthy group was calculated using the robust bootstrap method according to published guidelines (23). Additionally, the horn method for outlier detection was applied in the analysis. The Wilcoxon rank-sum two-tailed test was used to assess differences in L-lactate levels between CSF and blood in the healthy cattle group. Correlations between CSF L-lactate levels and variables (i.e., blood L-lactate, CSF red blood cells count [RBCC], CSF TNCC, CSF TP, CSF total neutrophil count, CSF total lymphocyte count, CSF total macrophage count) were examined for the sick animal group. All correlations were assessed with Spearman’s rank correlation coefficient. The Wilcoxon rank-sum two-tailed test was conducted to investigate differences in CSF and blood L-lactate levels between sick animals diagnosed with infectious-inflammatory disease (INF subgroup) and those without this disease (NON INF subgroup). Furthermore, the Kruskal-Wallis test and pairwise Mann–Whitney tests were employed to explore differences in CSF and blood L-lactate levels based on the variables derived from the VITAMIN D mnemonic. The same tests were performed to evaluate differences in CSF L-lactate, blood L-lactate, CSF TNCC, and CSF TP, and the final interpretation of CSF. The Benjamini-Hochberg procedure was used to adjust the *p*-values. Finally, receiver operating characteristic (ROC) analysis compared the levels of CSF L-lactate between cases of neutrophilic pleocytosis and those with other characteristics. The Youden index was employed to determine the optimal cut-off point. The odds ratio (OR) was calculated to assess the likelihood that elevated CSF L-lactate is associated with neutrophilic pleocytosis. Statistical significance was set at *p* < 0.05.

## 3 Results

### 3.1 Healthy cattle group

#### 3.1.1 Description

The healthy group was composed of 27 cattle: 9/27 (33%) females and 18/27 (67%) males. Most (24/27, 89%) were 7 months old, 2 were 6 months old, and 1 was 5 months old. All were Holstein Friesian breed. The median concentration of CSF TP was 38 mg/dL (IQR 30–46 mg/dL). The median CSF TNCC was 4.6 cells/μL (IQR 3.7–6.1 cells/μL). The median CSF RBCC was 30 cells/μL (IQR 10–385 cells/μL). The mean CSF L-lactate concentration was 1.7 mmol/L ± 0.4 mmol/L. Blood L-lactate concentration for all animals was known; the median concentration was 0.7 mmol/L (IQR 0.6–0.85 mmol/L).

#### 3.1.2 Reference intervals for L-lactate levels

**Figure 1:**
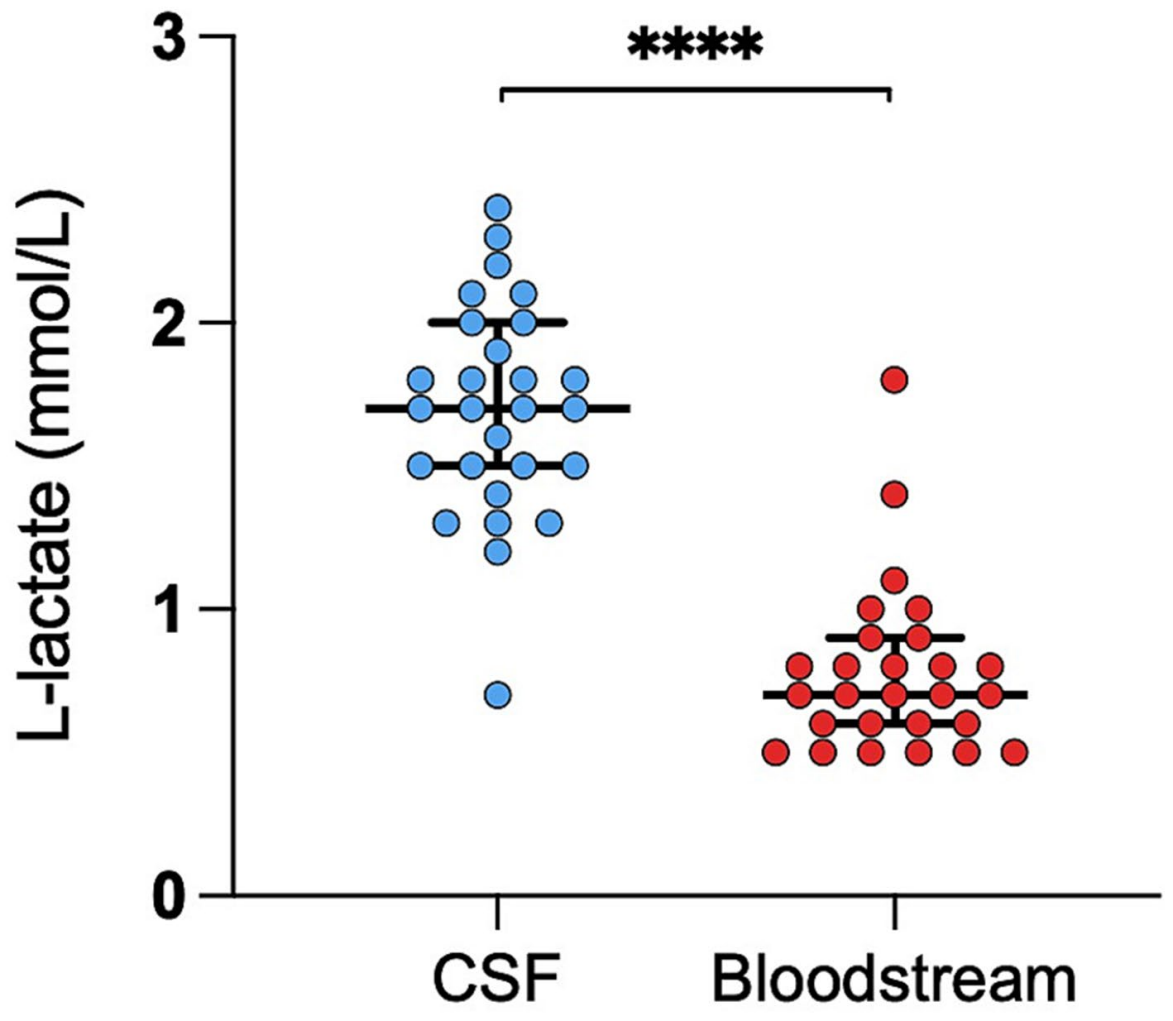
Difference in L-lactate levels between CSF and blood in healthy cattle. The scatter points represent individual data points. The median and IQR are shown. Asterisks (*) indicate the level of statistical significance: *p < 0.05, **p < 0.01, ***p < 0.001, ****p < 0.0001. CSF denotes cerebrospinal fluid.

The RI for CSF L-lactate was 1.1–2.4 mmol/L (lower CI 0.8–1.2; upper CI of 2.1–2.6). The RI for blood L-lactate was 0.2–1.2 mmol/L (lower CI 0–0.4; upper CI 1–1.4). No outliers were identified in either analysis. The median CSF L-lactate level was significantly higher than that of blood L-lactate (*p* < 0.0001; Figure 1).

### 3.2 Sick cattle group

#### 3.2.1 Description

The sick cattle group was composed of 86 neurologically impaired cattle: 44/86 (51%) females and 42/86 (49%) males. The median age was 20 days (IQR 6–180 days). The most frequent breed was Piemontese (76/86, 89%), followed by Holstein Friesian (7/86, 8%), mixed breed (2/86, 2%), and Limousine 1/86 (1%). The most frequent neuroanatomical localization was the forebrain (24/86, 28%) or multifocal localization (15/86, 17%). Brainstem involvement was observed in 9/86 (10%), a diffuse intracranial disorder in 9/86 (10%), L4-S3 spinal segment localization in 8/86 (9%), central vestibular system localization in 6/86 (7%), cerebellum involvement in 5/86 (6%), C6-T2 spinal segment involvement in 4/86 (5%), C1-C5 spinal segment involvement in 3/86 (4%), and T3-L3 spinal segment involvement in 3/86 (4%). The median concentration of CSF TP was 46 mg/dL (IQR 25–98 mg/dL). The median CSF TNCC was 7.8 cells/μL (IQR 2–65 cells/μL), and the median CSF RBCC was 80 cells/μL (15–1,335 cells/μL). CSF interpretation was normal in 40/86 (46%). Among the remaining cases, 24/86 (28%) exhibited mononuclear pleocytosis, 14/86 (16%) neutrophilic pleocytosis, 5/86 (6%) albuminocytologic dissociation, and 3/86 (4%) mixed pleocytosis, which refers to the presence of both mononuclear cells and neutrophils. The median CSF L-lactate level was 2.5 mmol/L (IQR 2–3.6 mmol/L). Blood L-lactate concentration was known for 60/86 cattle and the median level was 1.7 mmol/L (IQR 1.1–2.6 mmol/L). Over half (47/86, 55%) were diagnosed with an infectious- inflammatory CNS disorder (INF subgroup), while the remaining 39/86 (45%) had a CNS disorder of other origin (NON INF subgroup). Supplementary Table S1 outlines the etiological diagnosis for the INF subgroup. Within the NON INF subgroup, 15/39 cattle (38%) were diagnosed with an anomalous congenital condition, 12/39 (31%) with a metabolic-toxic disorder, 9/39 (23%) had experienced trauma, and 3/39 (8%) had a vascular disorder.

#### 3.2.2 Diagnostic findings of CSF L-lactate

**Figure 2:**
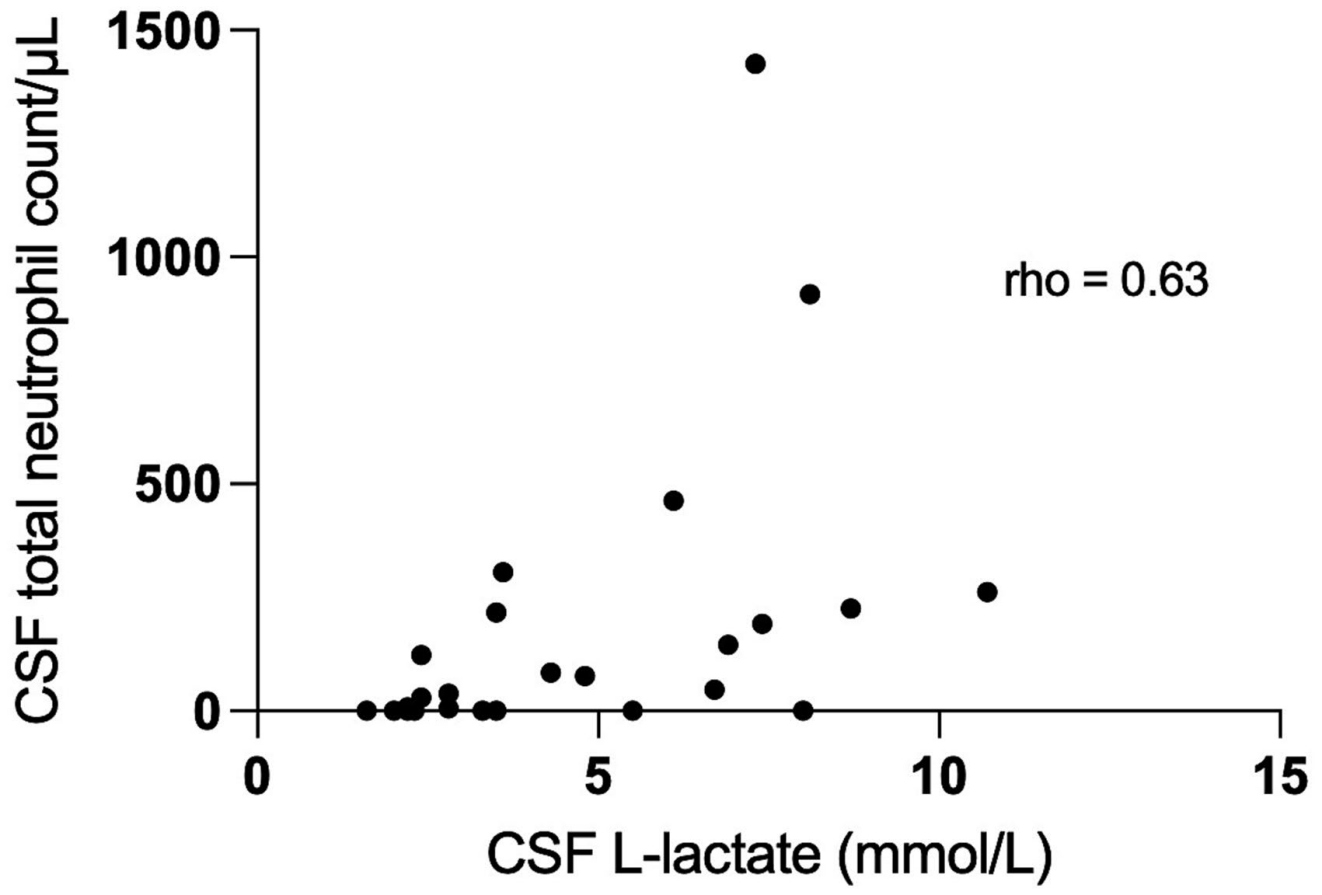
Correlation between CSF total neutrophil count/μL and CSF L-lactate levels in sick cattle. The scatter points represent individual data points. The X-axis represents CSF L-lactate and the Y-axis represents total neutrophil count. The correlation coefficient (rho 0.63; *p* = 0.0006) indicates the strength and the direction of the correlation between the variables. CSF denotes cerebrospinal fluid.

**Figure 3:**
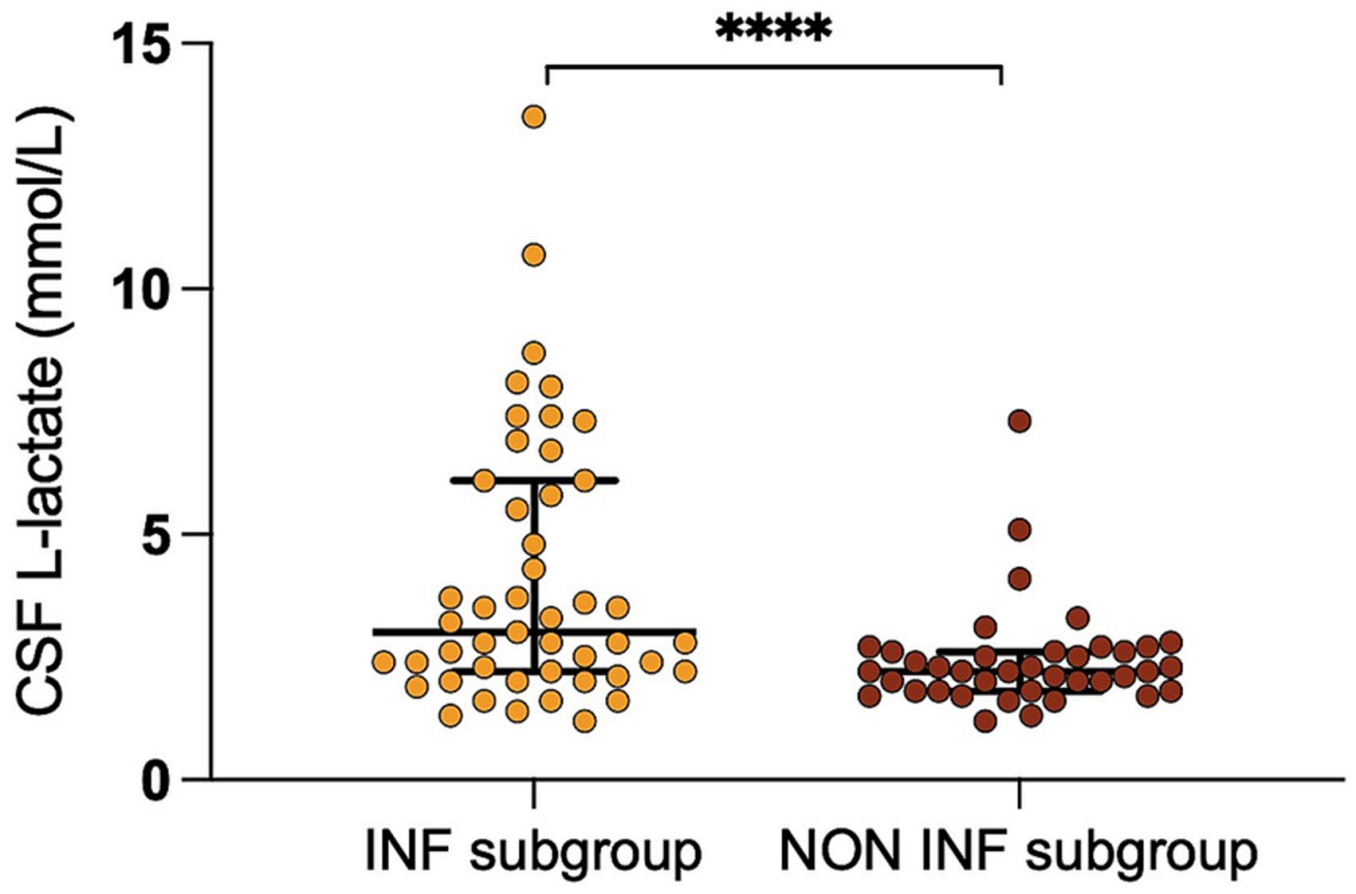
Difference in CSF L-lactate levels between the INF and the NON INF subgroup. The scatter points represent individual data points. The median and IQR are shown. Asterisks (*) indicate the level of statistical significance: *p < 0.05, **p < 0.01, ***p < 0.001, ****p < 0.0001. CSF denotes cerebrospinal fluid; INF subgroup, group with infectious-inflammatory CNS disorders; NON INF subgroup, group with noninfectious-noninflammatory CNS disorders.

**Figure 4:**
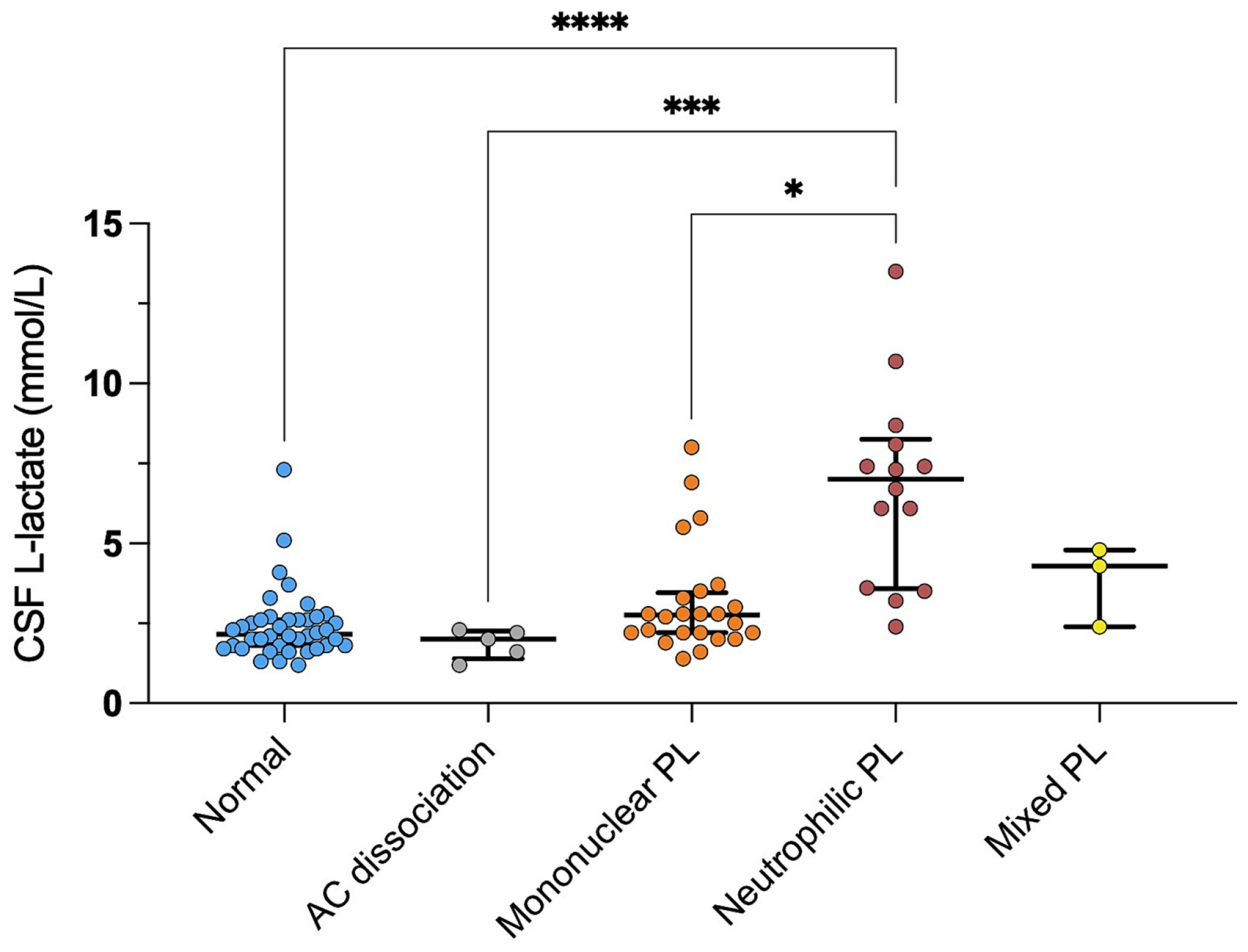
Difference in CSF L-lactate levels between CSF final interpretations in sick cattle. The scatter points represent individual data points. The median and IQR are shown. Asterisks (*) indicate the level of statistical significance: *p < 0.05, **p < 0.01, ***p < 0.001, ****p < 0.0001. The absence of an asterisk indicates a lack of statistically significant differences between groups. CSF denotes cerebrospinal fluid; AC. dissociation, albuminocytologic dissociation; mononuclear pl., mononuclear pleocytosis; neutrophilic pl., neutrophilic pleocytosis; mixed pl., mixed pleocytosis.

We found a significant correlation between CSF L-lactate concentration and the following variables in the sick cattle group: blood L-lactate (known for 60/86, rho = 0.34, *p* = 0.007, Supplementary Figure S1); CSF TNCC (rho = 0.57, *p* < 0.0001; Supplementary Figure S2); CSF TP (rho = 0.45, *p* < 0.0001; Supplementary Figure S3); CSF total neutrophil count (known for 25/86, rho = 0.63, *p* = 0.0006; Figure 2); CSF total macrophage count (known for 21/86, rho = 0.66, *p* = 0.001; Supplementary Figure S4). There was no significant correlation between CSF L-lactate and CSF RBCC or CSF total lymphocyte count (the latter known for 21/86). There was a statistically significant difference in CSF L-lactate levels between the INF and the NON INF subgroup (median CSF L-lactate INF subgroup 3 mmol/L, IQR 2.2–5.95 mmol/L; median CSF L-lactate NON INF subgroup 2.2 mmol/L, IQR 1.8–2.6 mmol/L; *p* < 0.0001; Figure 3). However, when the INF subgroup and each of the other VITAMIN D categories were compared and the *p*-values corrected, this significant difference disappeared (Supplementary Figure S5). There were no significant differences in blood L-lactate levels across the various diagnoses (Supplementary Figures S6, S7). In contrast, significant differences were observed in CSF L-lactate levels, CSF TNCC/μL, and CSF TP levels for the final interpretations of CSF (*p* < 0.0001), with the highest CSF L-lactate, CSF TNCC, and CSF TP found in neutrophilic pleocytosis cases. The characteristics of these variables in each CSF interpretation, along with the *p*-values obtained from pairwise comparisons, are presented in Tables 1–3; the corresponding results are illustrated in Figure 4 and Supplementary Figures S8, S9. In contrast, no significant differences in blood L-lactate levels were observed across interpretations of CSF (Supplementary Figure S10).

**Table 1:** Summary characteristics of CSF L-lactate levels in the sick cattle for each CSF interpretation and the results of pairwise multiple comparison.

	Median CSF L-lactate mmol/L (IQR)	Normal	AC. dissociation	Mononuclear pl.	Neutrophilic pl.	Mixed pl.
Normal	2.15 (1.8–2.6)	-	0.20	0.05	< 0.0001	0.08
AC. dissociation	2 (1.6–2.2)	0.20	-	0.06	0.005	0.06
Mononuclear pl.	2.75 (2.2–3.35)	0.05	0.06	-	0.0006	0.30
Neutrophilic pl.	7 (4.22–7.92)	< 0.0001	0.005	0.0006	-	0.16
Mixed pl.	4.3 (3.35–4.55)	0.08	0.06	0.30	0.16	-

CSF denotes cerebrospinal fluid; AC. dissociation, albuminocytologic dissociation; mononuclear pl., mononuclear pleocytosis; neutrophilic pl., neutrophilic pleocytosis; mixed pl., mixed pleocytosis.

**Table 2:** Summary characteristics of CSF TNCC/μL in the sick cattle for each CSF interpretation and the results of pairwise multiple comparison.

	Median TNCC/μL (IQR)	Normal	AC. dissociation	Mononuclear pl.	Neutrophilic pl.	Mixed pl.
Normal	2 (1.2–4)	-	0.32	< 0.0001	< 0.0001	0.007
AC. dissociation	5.2 (1.8–5.4)	0.32	-	0.0003	0.0003	0.04
Mononuclear pl.	18.2 (11.72–45.65)	< 0.0001	0.0003	-	< 0.0001	0.02
Neutrophilic pl.	601.5 (272.2–1932)	< 0.0001	0.0003	< 0.0001	-	0.04
Mixed pl.	166.2 (123.4–193.6)	0.007	0.04	0.02	0.04	-

CSF denotes cerebrospinal fluid; TNCC, total nucleated cell count; AC. dissociation, albuminocytologic dissociation; mononuclear pl., mononuclear pleocytosis; neutrophilic pl., neutrophilic pleocytosis; mixed pl., mixed pleocytosis.

**Table 3:** Summary characteristics of CSF TP levels in the sick cattle for each CSF interpretation and the results of pairwise multiple comparison.

	Median TP mg/dL (IQR)	Normal	AC. dissociation	Mononuclear pl.	Neutrophilic pl.	Mixed pl.
Normal	25 (20–32)	-	0.006	0.0002	< 0.0001	0.01
AC. dissociation	62 (53–62)	0.006	-	0.35	0.006	0.04
Mononuclear pl.	50 (37.75–72.5)	0.0002	0.35	-	0.0002	0.03
Neutrophilic pl.	267 (223–348)	< 0.0001	0.006	0.0002	-	0.36
Mixed pl.	178 (167–250)	0.01	0.04	0.03	0.36	-

CSF denotes cerebrospinal fluid; TP, total microprotein; AC. dissociation, albuminocytologic dissociation; mononuclear pl., mononuclear pleocytosis; neutrophilic pl., neutrophilic pleocytosis; mixed pl., mixed pleocytosis.

**Figure 5:**
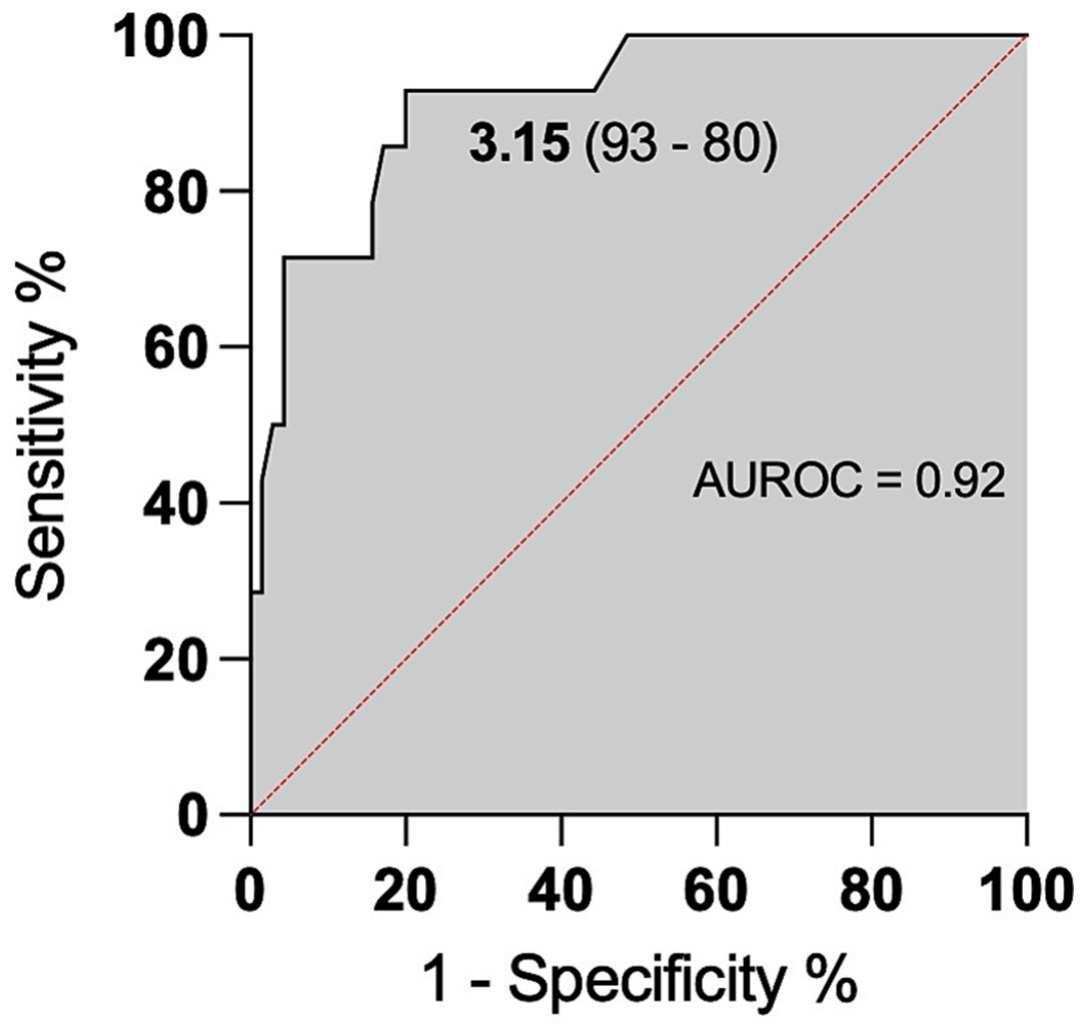
ROC curve for predicting neutrophilic pleocytosis based on CSF L-lactate levels. The x-axis represents the false positive rate (1-Specificity), and the y-axis represents the true positive rate (Sensitivity). The area under the ROC curve is 0.92, indicating the model’s discriminative ability. The CSF L-lactate cut-off of 3.15 mmol/L is the threshold for classifying cases as positive or negative for neutrophilic pleocytosis. The model had a sensitivity of 93%, correctly identifying 93% of cases with neutrophilic pleocytosis, and a specificity of 80%, correctly identifying 80% of cases without neutrophilic pleocytosis.

We observed higher levels of CSF L-lactate in the cases of neutrophilic pleocytosis and analyzed the receiver operating characteristic (ROC) curve. The area under the ROC curve (AUROC) for CSF L-lactate concentration in the neutrophilic pleocytosis group versus the other groups was 0.92. The Youden index method identified a threshold of 3.15 mmol/L for CSF L-lactate, which yielded a diagnostic sensitivity of 93% and a specificity of 80% in the detection of neutrophilic pleocytosis (Figure 5). Cattle with CSF L-lactate levels above the threshold were 54 times more likely to exhibit neutrophilic pleocytosis compared to those with normal CSF L-lactate levels (OR 53.86, 95% CI 6.49–446.91).

## 4 Discussion

To our best knowledge, this is the first study to determine a RI for CSF L-lactate in cattle. In a recent study, CSF L-lactate levels were evaluated in healthy cattle. However, the authors only reported the mean value ± SD of the examined samples and could not establish a proper RI for health due to the small sample size (19). We noted significantly higher L-lactate levels in the CSF than in the blood in the healthy cattle group. This observation is shared by Posner et al. (24) and further supports the notion that blood and CSF L-lactate levels are independent of each other. Several factors may contribute to this difference, including processes influencing the production and the clearance of L-lactate in the CSF. L-lactate is produced primarily by astrocytes in the brain in response to neuronal activity by aerobic glycolysis. This metabolic pathway is specific to astrocytes and is regulated by a gene expression profile that promotes L-lactate production from pyruvate rather than its utilization in the tricarboxylic acid cycle. This distinctive process might lead to elevated L-lactate production within the brain and subsequently in the CSF (25). Additionally, L-lactate clearance from the CSF may be slower than its clearance from the bloodstream, further contributing to the higher levels in CSF compared to blood (26).

When we compared the CSF L-lactate levels in the sick cattle group, we identified relatively weak differences between the INF and the NON INF subgroup. This is evident from the overlapping distribution of values between the two subgroups, with a few higher values in the INF subgroup contributing to the observed statistical significance (Figure 3). Elevated CSF L-lactate levels have been documented in humans with bacterial infections of the CNS, and cut-off values have been proposed as a way to distinguish them from aseptic inflammations (11). Based on this notion, we sought a more accurate method to differentiate bacterial infections, particularly in the acute phase, from other disorders. Following this idea, we observed significant differences in L-lactate levels together with alterations in the CSF. Notably, the highest L-lactate levels were observed in the subjects with neutrophilic pleocytosis, and a CSF L-lactate level above 3.15 mmoL/L was highly predictive of this condition.

Neutrophilic pleocytosis is commonly encountered in acute bacterial infection of the CNS in cattle and it is considered specific for such disease. Neutrophils are the first cells to be recruited to the site of infection where they participate in anti-microbial host defense as regulators of both innate and adaptive immunity. Activated neutrophils display diverse effector mechanisms, including phagocytosis, activation of NADPH oxidase, release of granular contents, and release of neutrophil extracellular traps (NETs). These NETs consist of DNA, histones, microbicidal peptides, and antimicrobial enzymes and are associated with L-lactate formation via the Warburg effect (27–29). Accordingly, we examined the relationship between total neutrophilic count and CSF L-lactate levels and found a moderate-to-strong correlation between the two. Although our analysis was performed on a small subgroup, potentially limiting the external validity of the results, our findings align with studies in human medicine (27–29). Of note, our study does not provide conclusive evidence about whether L-lactate levels are more closely linked to the functional state of neutrophils or their absolute numbers. Moreover, we found similar correlations between total macrophage count and L-lactate levels, which suggests that L-lactate might be secondary to general differences in CNS inflammation. Further studies are needed to develop this hypothesis and determine the production rate of L-lactate from neutrophils and other immune cells isolated from the CSF in relation to their activation status. Significant correlations were observed between CSF L-lactate concentration and CSF TNCC and TP. The cases of neutrophilic pleocytosis exhibited elevated TNCC and TP levels, which might explain the correlation. Additional studies are needed to investigate the functional relationship between these variables and CSF L-lactate concentration.

We did not to exclude samples with blood contamination since it is widely believed in both human and veterinary medicine that iatrogenic blood contamination of the CSF does not alter L-lactate concentration (15, 17, 30, 31). Our results further support this notion, as no correlation was observed between CSF L-lactate levels and RBCC.

We noted a weak correlation between CSF L-lactate and blood L-lactate levels in the sick cattle group. This finding contradicts the current literature, which states that CSF and blood L-lactate levels are independent of one another (24, 32). A plausible explanation for our observation is that the integrity of the blood–brain barrier may be altered in disease, with a partial effect of blood L-lactate concentration on CSF L-lactate concentration (12, 13). No significant differences in blood L-lactate levels were observed across diagnoses or CSF interpretations. This underlines the importance of measuring L-lactate in CSF rather than in blood to accurately differentiate acute bacterial infections of the CNS.

This study has some limitations. There were differences in the demographics between the healthy cattle, on which the RI of CSF L-lactate was calculated, and the sick cattle, particularly for age and breed. The healthy group consisted exclusively of young Holstein Friesian cattle. Slight differences in the RI of CSF L-lactate have been reported between human neonates and adults (33). Caution is warranted when applying the RI obtained in this study to evaluate the CSF L-lactate levels in animals differing in age, as it may not be directly applicable. Further studies are needed to evaluate the RI of lactate in both neonatal and adult healthy cattle, as well as to assess the impact of different breed usage (dairy, beef, or dual-purpose).

The bacterial agents of infection were not identified, which precluded investigating correlations between specific types of bacteria and lactate levels. Bacterial metabolism is known to play a relatively minor role in lactate production, with the host’s immune response being the primary determinant (28). Furthermore, our focus was solely on the measurement of L-lactate, without taking into account the other isotype, D-lactate, which is produced by bacteria (34). This decision was based on the fact that D-lactate is typically found in low concentrations, accounting for 1–5% of L-lactate (34). Commonly used lactate measurement methods primarily target L-lactate.

In conclusion, our findings underscore a strong association between CSF L-lactate levels and neutrophilic pleocytosis. This can be interpreted as a marker of acute CNS inflammation induced by bacterial infection in cattle. A CSF L-lactate cut-off value of 3.15 mmol/L for identifying this kind of disease appears therefore reasonable. Measuring CSF L-lactate in field conditions requires only a small volume of CSF, less than one drop, and yields results within a few seconds. The quick turnaround time, along with the high sensitivity and specificity in detecting acute inflammatory disorders, can provide veterinarians with the advantage of obtaining prompt results and the opportunity to initiate antimicrobial treatments as needed, thus promoting a more rational use of antimicrobial drugs in farm animal practice.

## Data Availability

The raw data supporting the conclusions of this article will be made available by the authors, without undue reservation.
