# Supplementary material for: Cerebrospinal fluid L-lactate as a diagnostic marker for infectious-inflammatory disorders in the central nervous system of cattle
Source: Front Vet Sci. 2024 Oct 9;11:1466920. doi: 10.3389/fvets.2024.1466920 (PMC11496040; doi:10.3389/fvets.2024.1466920)
Supplement: Supplementary file 1 [file Table_1.pdf]

Table S1. Distribution of infectious-inflammatory etiologies.

**Infectious-inflammatory conditions (INF subgroup)**<br>N=47	**Confirmed diagnosis (based on necropsy or antemortem CSF culture)**	**Suspected diagnosis (based on clinic-laboratory findings and response to specific treatment)**
**Listeriosis**<br>N =4	/	N = 4
**Neonatal bacterial meningitis/meningoencephalitis**<br>N = 21	N = 11	N = 10
**Non-neonatal bacterial meningitis/meningoencephalitis**<br>N = 12	N = 4	N = 8
**Otogenic meningoencephalitis**<br>N = 1	/	N= 1
**Undefined meningitis/meningoencephalitis**<br>N = 9	/	N = 9
