# Supplementary figures and images for: Cerebrospinal fluid L-lactate as a diagnostic marker for infectious-inflammatory disorders in the central nervous system of cattle
Source: Front Vet Sci. 2024 Oct 9;11:1466920. doi: 10.3389/fvets.2024.1466920 (PMC11496040; doi:10.3389/fvets.2024.1466920)

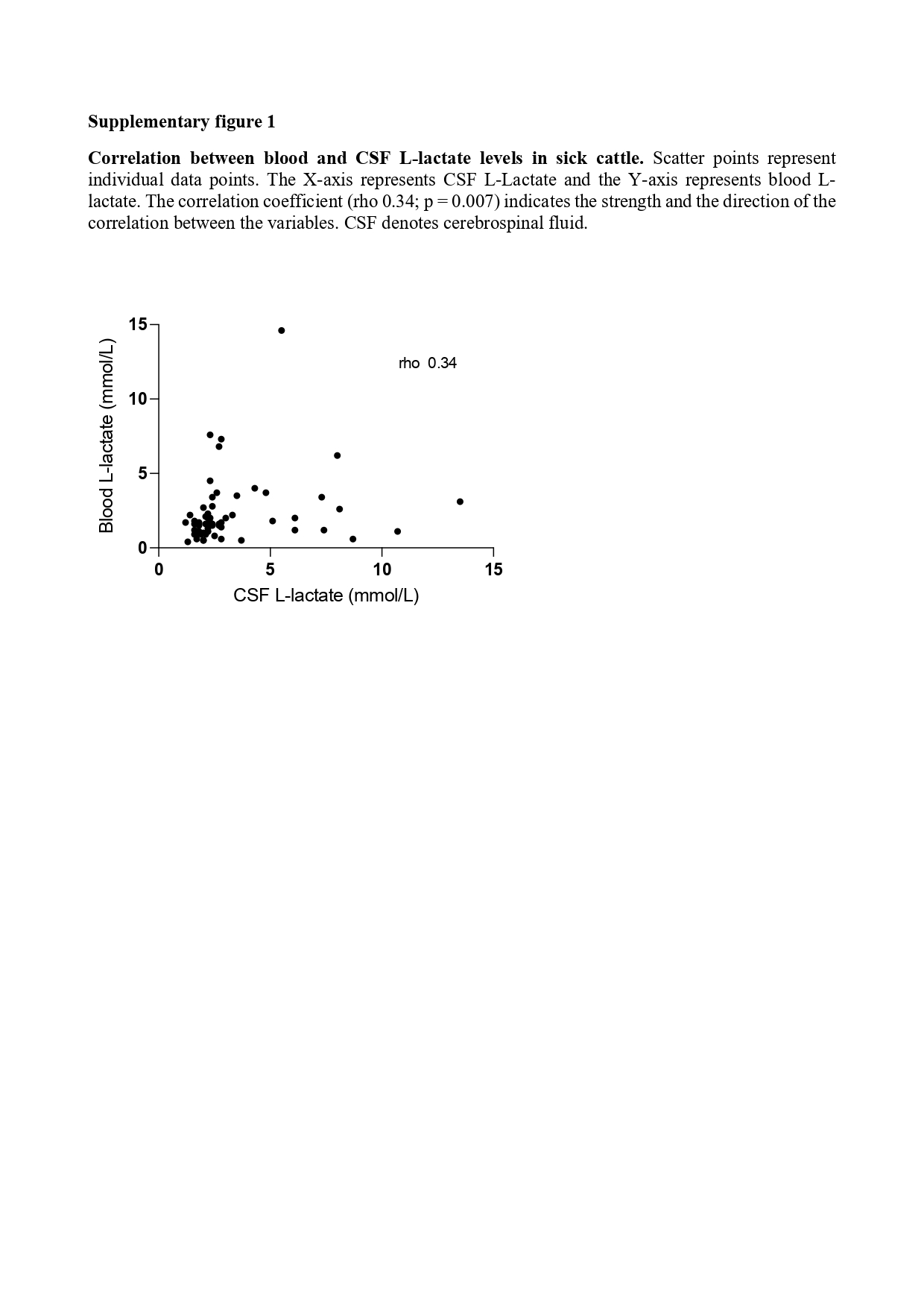

Supplement: Supplementary file 2 [file Image_1.jpg]

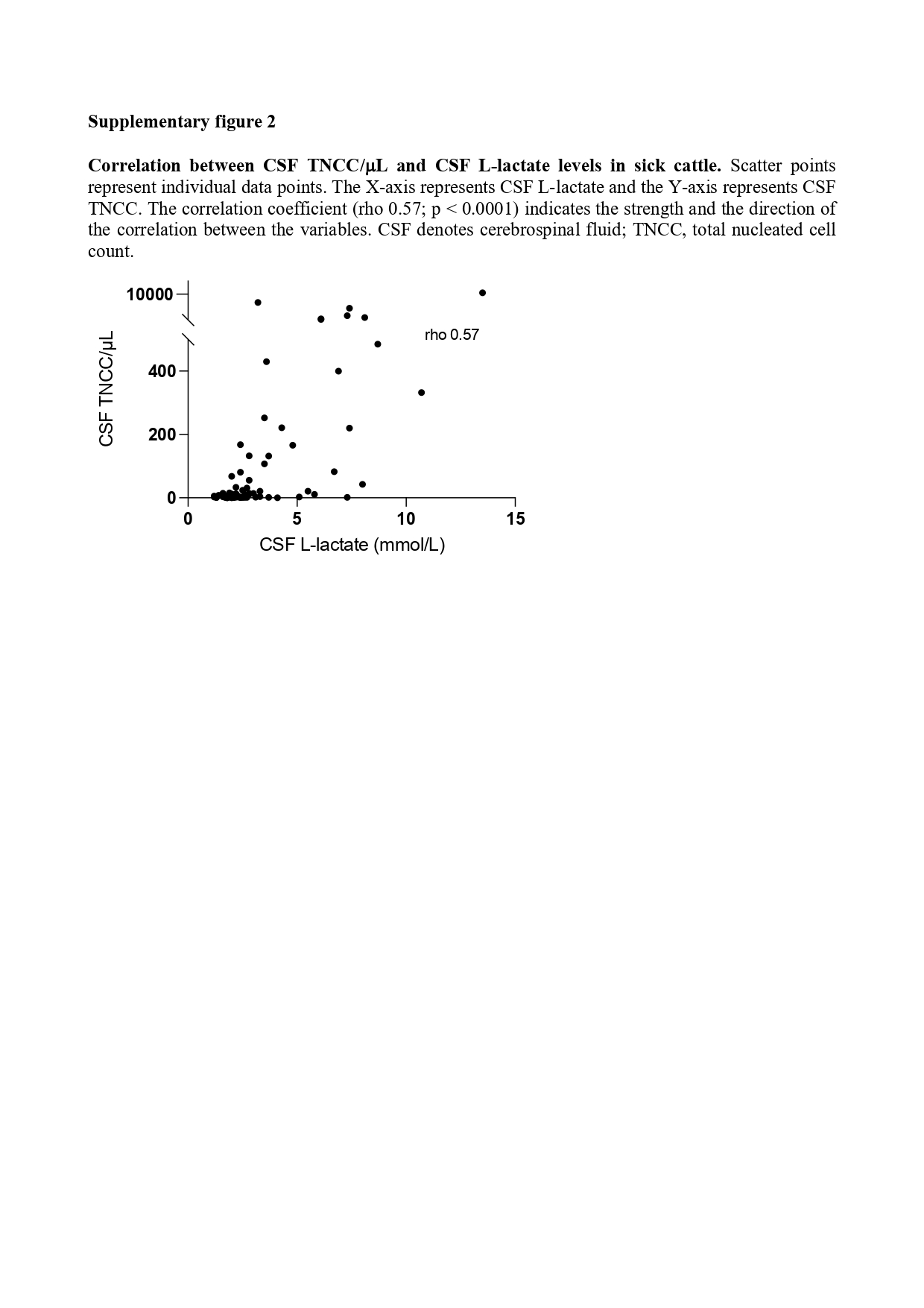

Supplement: Supplementary file 3 [file Image_2.jpg]

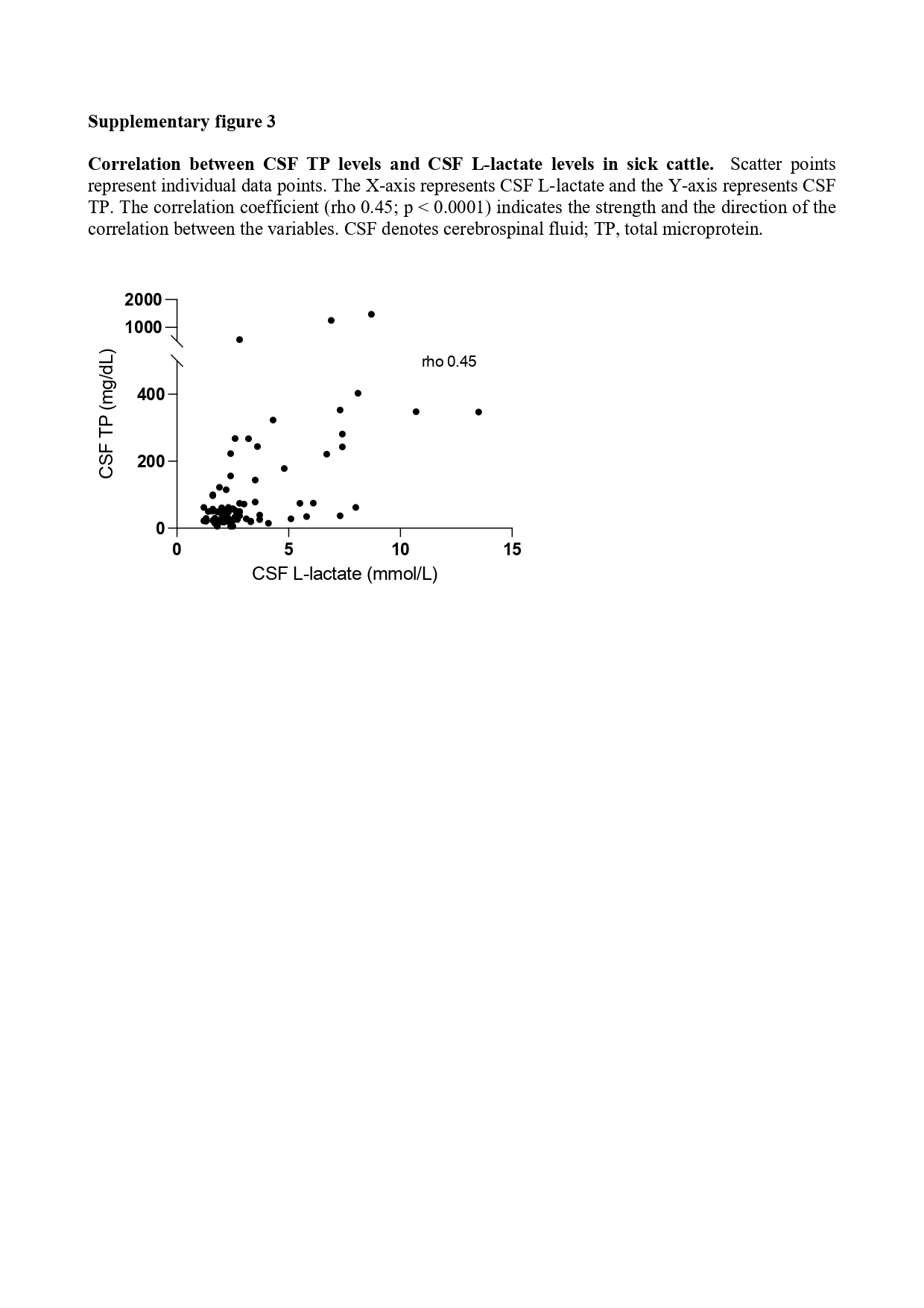

Supplement: Supplementary file 4 [file Image_3.jpg]

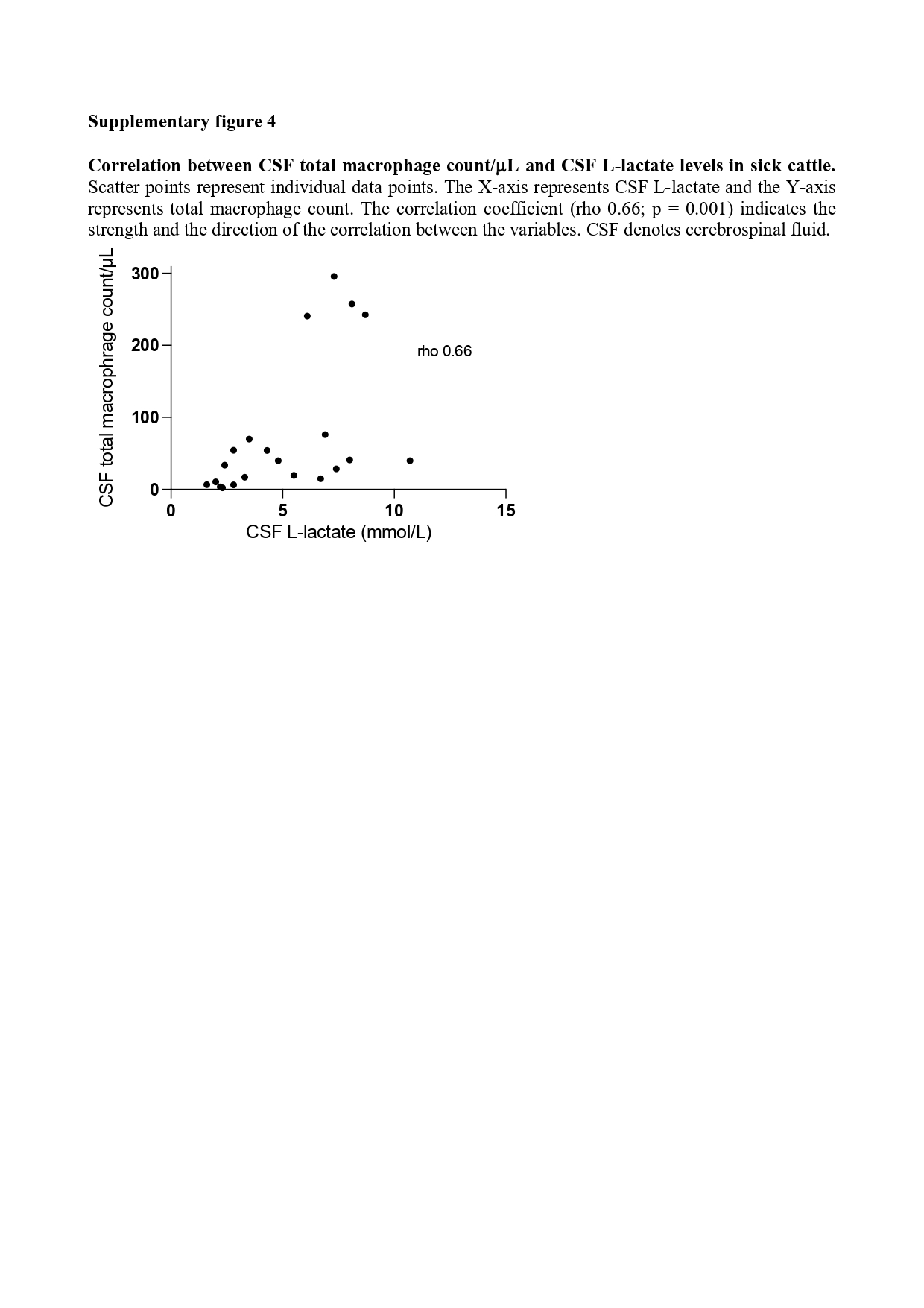

Supplement: Supplementary file 5 [file Image_4.jpg]

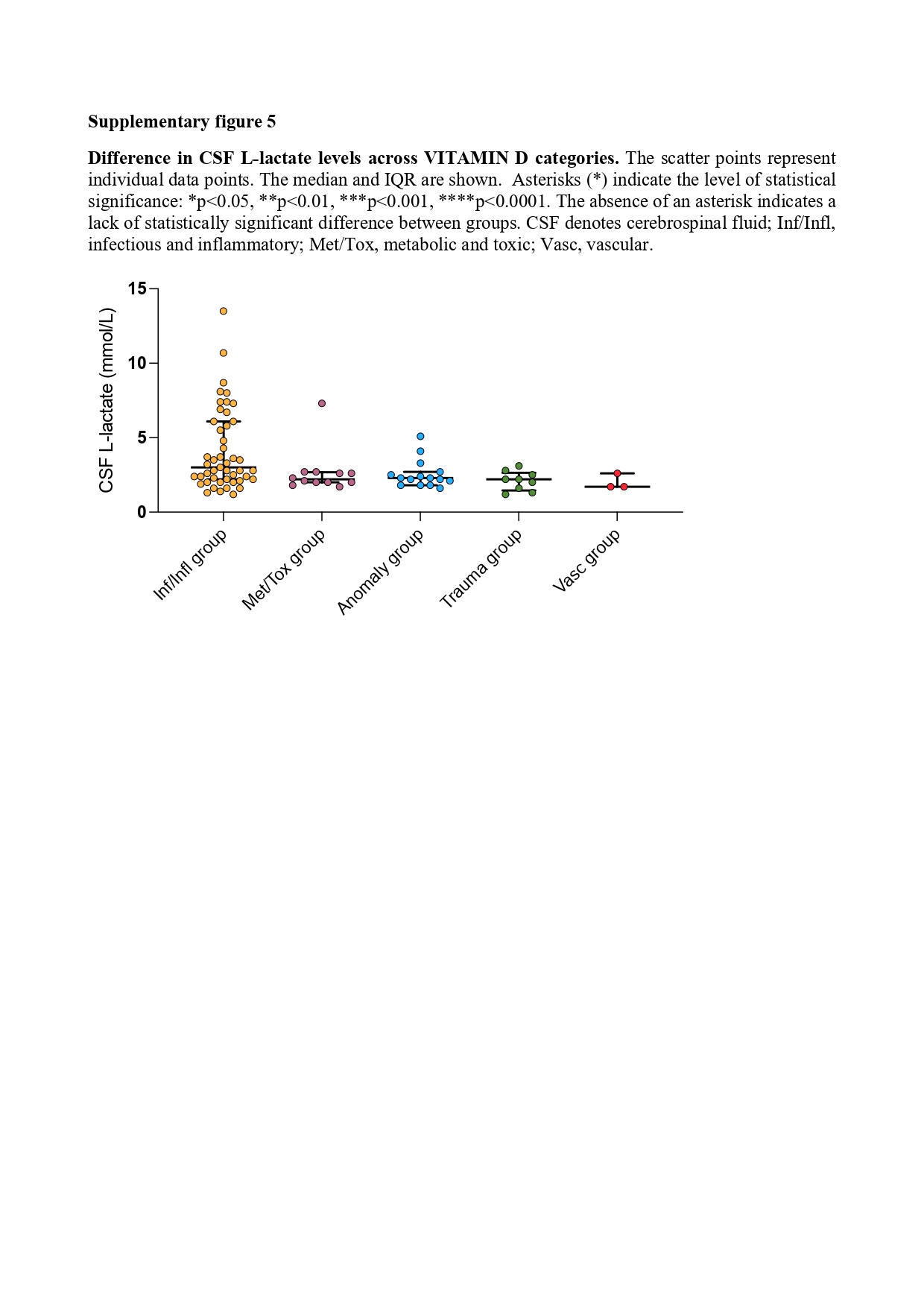

Supplement: Supplementary file 6 [file Image_5.jpg]

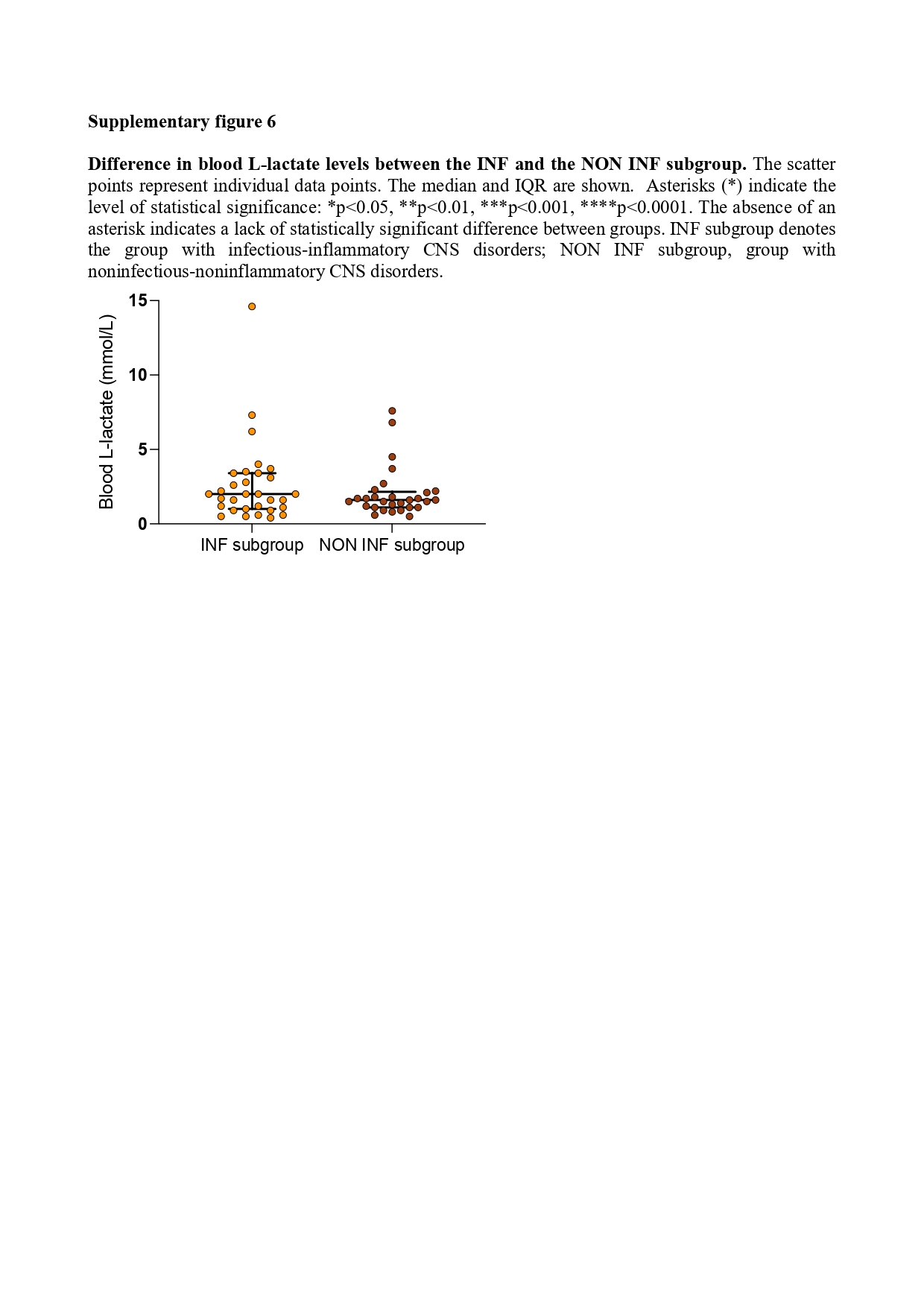

Supplement: Supplementary file 7 [file Image_6.jpg]

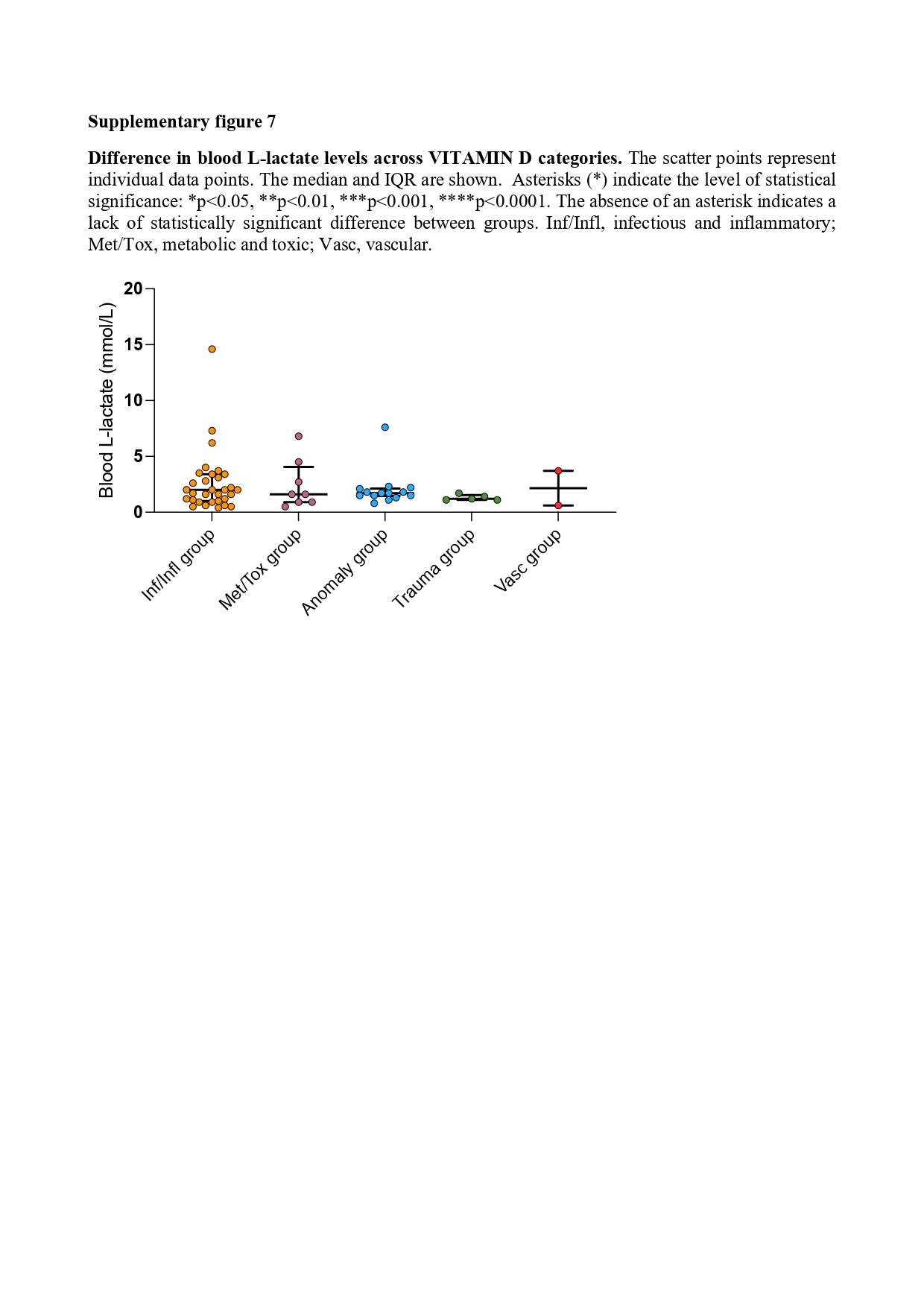

Supplement: Supplementary file 8 [file Image_7.jpg]

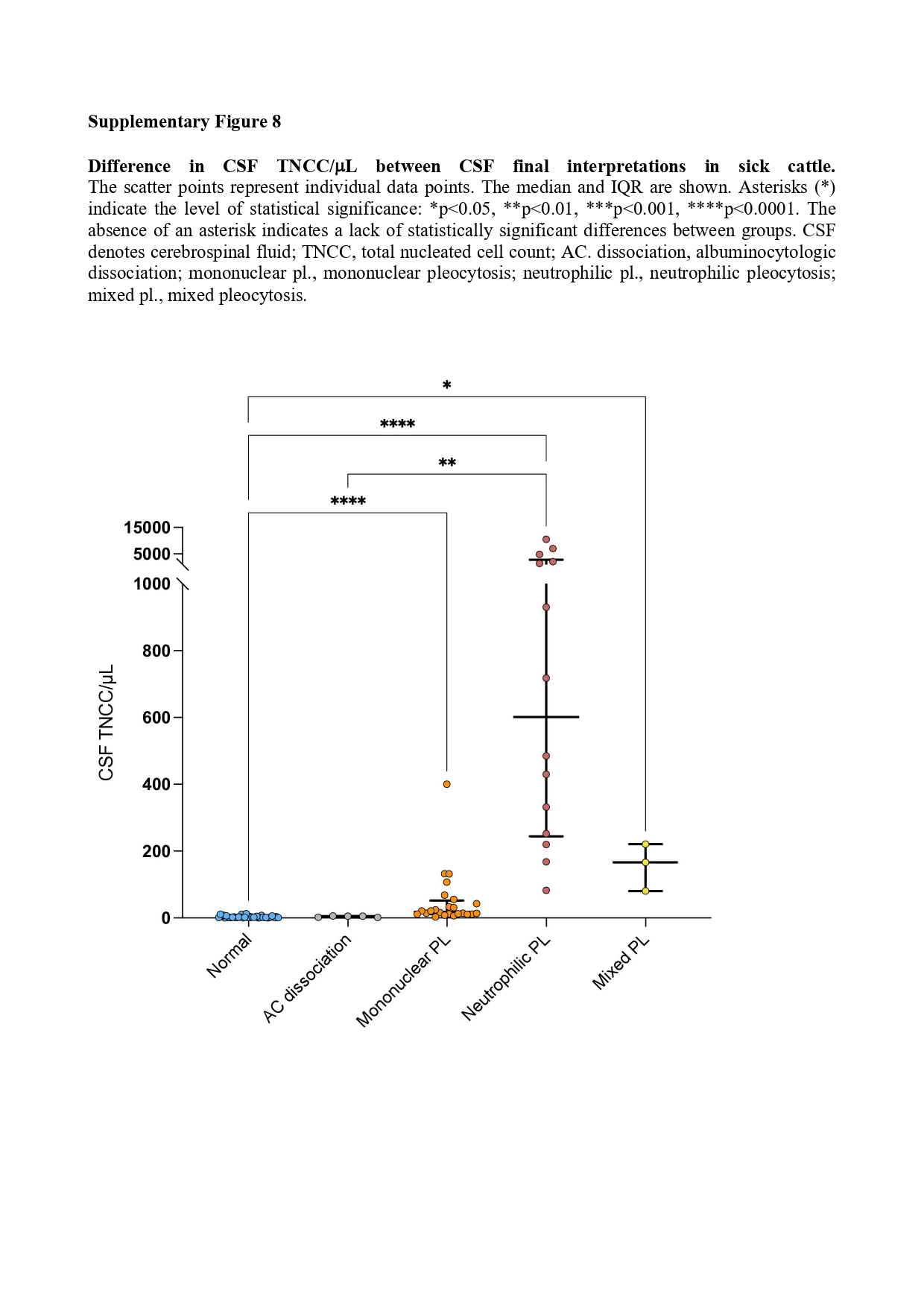

Supplement: Supplementary file 9 [file Image_8.jpg]

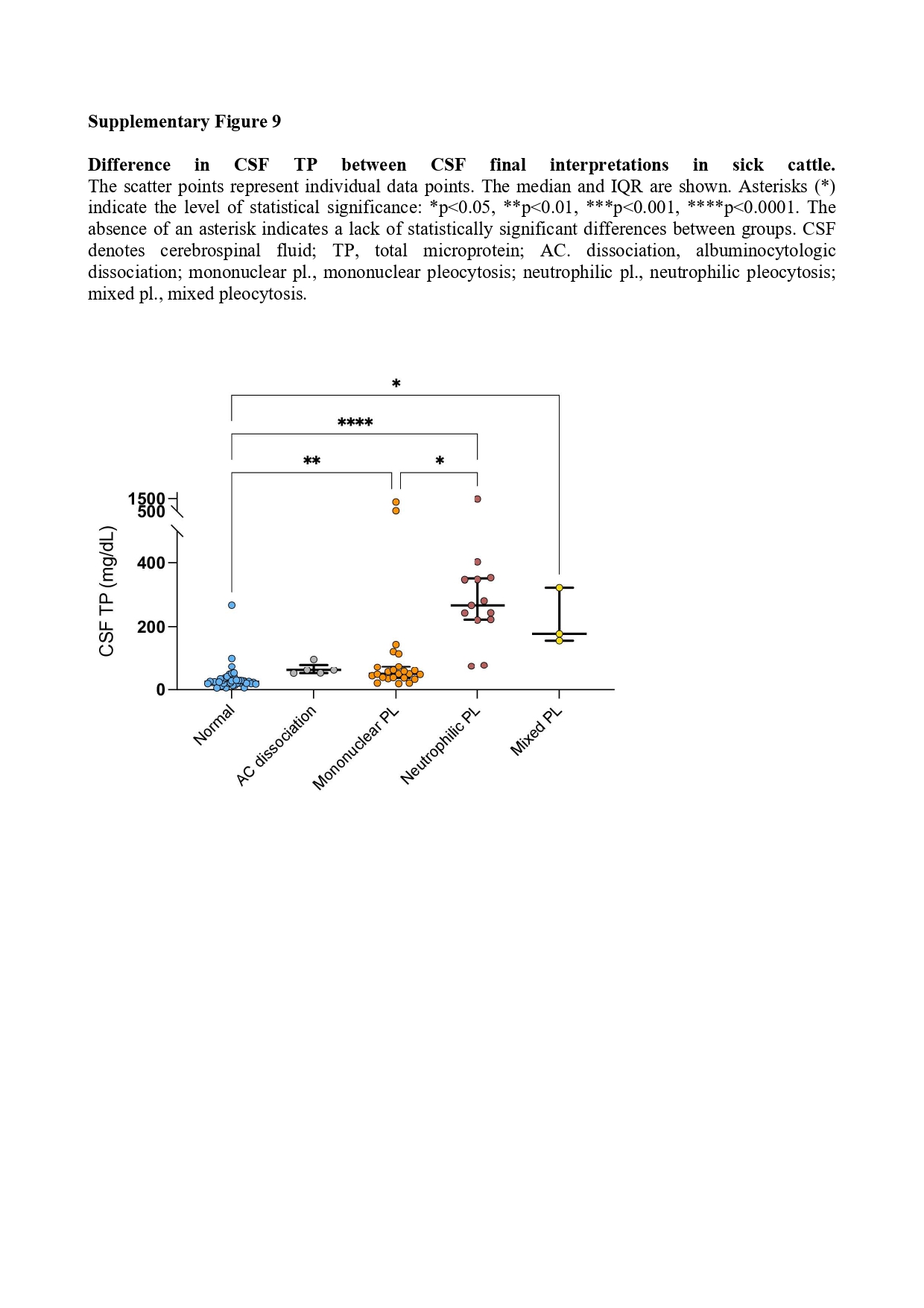

Supplement: Supplementary file 10 [file Image_9.jpg]

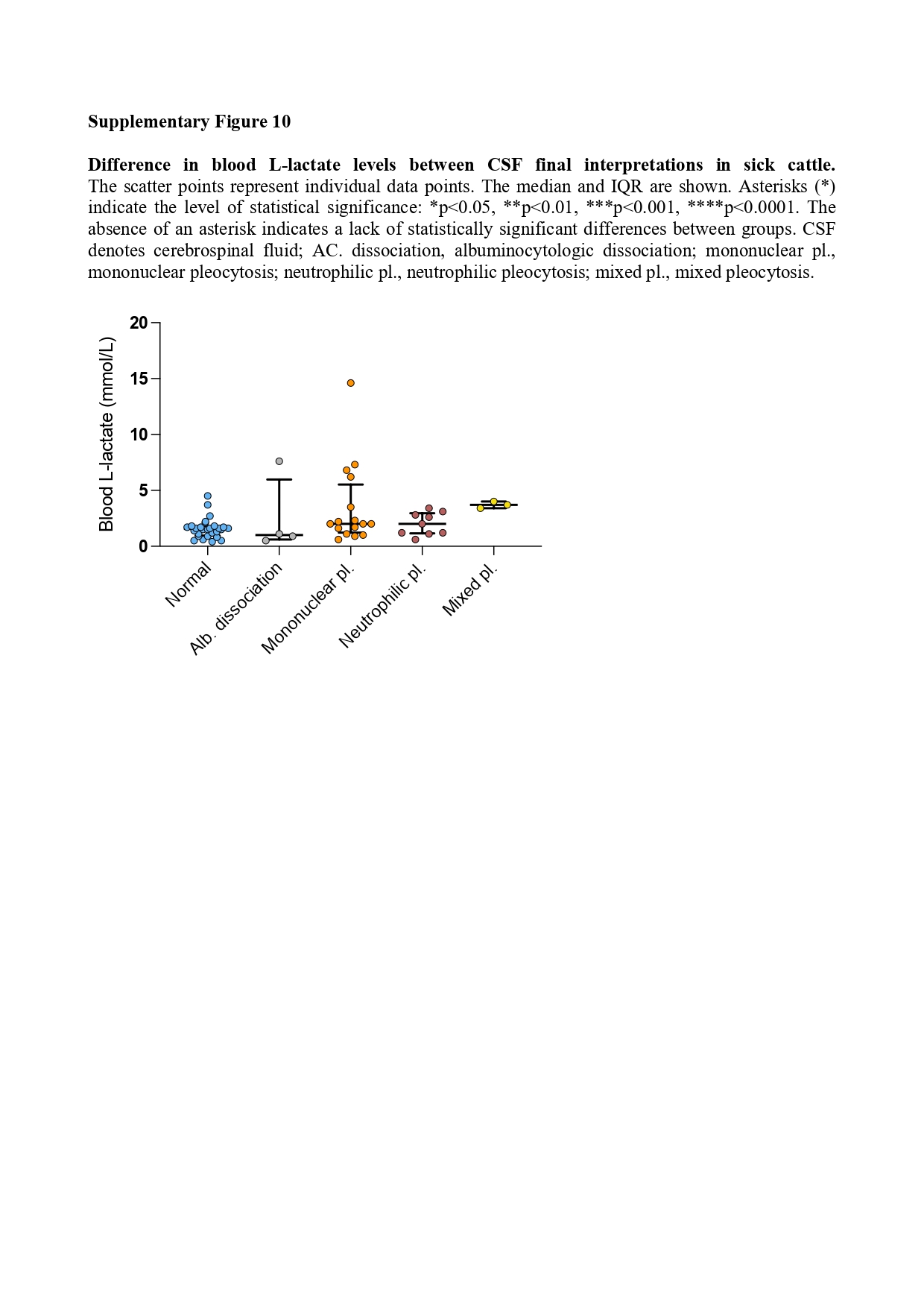

Supplement: Supplementary file 11 [file Image_10.jpg]
